# First detection of *Borrelia miyamotoi* in *Ixodes ricinus* ticks from northern Italy

**DOI:** 10.1186/s13071-018-2713-z

**Published:** 2018-03-20

**Authors:** Silvia Ravagnan, Laura Tomassone, Fabrizio Montarsi, Aleksandra Iwona Krawczyk, Eleonora Mastrorilli, Hein Sprong, Adelaide Milani, Luca Rossi, Gioia Capelli

**Affiliations:** 1 Istituto Zooprofilattico Sperimentale delle Venezie, Legnaro, Italy; 2 Dipartimento Scienze Veterinarie, Università degli Studi di Torino, Grugliasco, Italy; 3 National Institute of Public Health and Environment (RIVM), Bilthoven, The Netherlands

**Keywords:** *Borrelia miyamotoi*, *Ixodes ricinus*, Zoonosis, Northern Italy

## Abstract

**Background:**

*Borrelia miyamotoi* is a spirochete transmitted by several ixodid tick species. It causes a relapsing fever in humans and is currently considered as an emerging pathogen. In Europe, *B. miyamotoi* seems to occur at low prevalence in *Ixodes ricinus* ticks but has a wide distribution. Here we report the first detection of *B. miyamotoi* in *Ixodes ricinus* ticks collected in two independent studies conducted in 2016 in the north-eastern and north-western Alps, Italy.

**Results:**

Three out of 405 nymphs (0.74%) tested positive for *Borrelia miyamotoi*. In particular, *B. miyamotoi* was found in 2/365 nymphs in the western and in 1/40 nymphs in the eastern alpine area. These are the first findings of *B. miyamotoi* in Italy.

**Conclusions:**

Exposure to *B. miyamotoi* and risk of human infection may occur through tick bites in northern Italy. Relapsing fever caused by *Borrelia miyamotoi* has not yet been reported in Italy, but misdiagnoses with tick-borne encephalitis, human granulocytic anaplasmosis or other relapsing fever can occur. Our findings suggest that *B. miyamotoi* should be considered in the differential diagnosis of febrile patients originating from Lyme borreliosis endemic regions. The distribution of this pathogen and its relevance to public health need further investigation.

## Background

*Borrelia miyamotoi* is a spirochete transmitted to vertebrate hosts by the same hard ticks that transmit *Borrelia burgdorferi* (*sensu lato*), the agent of Lyme disease, namely *Ixodes ricinus* and *I. persulcatus* in Europe, *I. persulcatus*, *I. ovatus* and *I. pavlovskyi* in Asia, and *I. scapularis* and *I. pacificus* in the USA [1]. *Borrelia miyamotoi* was first identified in 1994 in ticks from Japan [2] and is currently considered an emerging pathogen affecting humans, in whom it can cause an infection similar to a relapsing fever [1, 3, 4]. Three types of *B. miyamotoi* are currently recognized: American, Asian (Siberian) and European.

In Europe, wild rodents are reservoir hosts for *B. miyamotoi* [5–7]. The pathogen has been shown to be widespread [5, 8], and occurs in *I. ricinus* at a low prevalence, with the highest frequency registered to date in Hungary (4.8%) [9].

Despite the increasing number of human cases in the recent years [5], our knowledge on the distribution, ecology and epidemiology of *B. miyamotoi* is limited. To date, *B. miyamotoi* had not been detected in humans or in *I. ricinus* ticks in Italy, probably because it had not been searched for. In fact, in northern Italy, as in other European countries, *I. ricinus* is the tick species that most frequently bites humans [10–12].

The Italian Alps in northern Italy, offer favourable environmental conditions for the survival and proliferation of *I. ricinus* [13], which were found to be infected by several tick-borne pathogens (TBPs), namely *B. burgdorferi* (*s.l.*), spotted fever group rickettsiae, “*Candidatus* Neoehrlichia mikurensis”, *Anaplasma phagocytophilum*, tick-borne encephalitis flavivirus, and *Babesia* spp. [14–20]. The eastern Alps have been considered a hot-spot for tick-borne human infections for a long time [21, 22], but the invasion of *I. ricinus* and associated pathogens in the western area is a more recent phenomenon [17].

In this short note, we report the first detection of *B. miyamotoi* in *I. ricinus* ticks collected in two independent studies conducted in 2016 in the north-eastern and north-western Alps of Italy.

## Methods

Ticks were collected by standard dragging, using a 1 m^2^ white flannel cloth. In the north-western alpine area, 45 sites at different altitudes [range 950–1880 m above sea level (masl)] were monitored in a regional natural park in Susa Valley, Turin Province (http://www.parchialpicozie.it/). Here, tick bites are increasingly reported by people visiting the protected area and cases of Lyme disease have recently been reported. The aim of the study was to explore the diversity, abundance and distribution of ticks within the park and determine the prevalence of TBPs. *Borrelia miyamotoi* was searched by a qPCR targeting a fragment of the *flagellin* gene [23] in a sample of 365 individually tested nymphs.

In the north-eastern alpine area, ticks were collected in eight sites located at different altitudes (range 324–1050 masl) in three areas recognized to be endemic for TBPs [16], namely Verona, Belluno and Udine provinces. The aim of this study was to characterize the microbiota of collected *I. ricinus*, using targeted amplicon sequencing (*16S* rDNA). Individual adults (17) and 10 pools of 4 nymphs each (*n* = 40) were examined. The high-throughput sequencing analysis identified a sequence belonging to *B. miyamotoi*. The presence of *B. miyamotoi* was then confirmed by a specific real time PCR targeting the *glpQ* gene [24].

To harmonize the results and for sequencing, one positive sample from the north-western area and the positive sample from the north-eastern area were further amplified by a traditional PCR targeting ~900 bp of the *glpQ* gene [23]. For the second positive sample from the north-western area the extracted DNA was insufficient for further amplification.

The PCR products of *glpQ* gene were sequenced with both forward and reverse primers using a 16-capillary ABI PRISM 3130xl Genetic Analyzer (Applied Biosystem, Foster City, CA, USA) and compared with representative sequences available in GenBank using the Basic Local Alignment Search Tool (BLAST). Phylogenetic analyses were carried out using the neighbour-joining (N-J) method, with 1000 bootstrap replicates implemented in the MEGA 6 programme [25].

Confidence intervals for prevalence have been calculated with the free software WinEpi available from: http://www.winepi.net/uk/index.htm.

## Results

Overall, three out of 405 *I. ricinus* nymphs (0.74%; 95% CI: 0.15–2.1%) tested positive for *B. miyamotoi*. In detail, *B. miyamotoi* was found in 2/365 nymphs (0.5%; 95% CI: 0.07–1.96%) originating from the western area and in 1/40 nymphs (2.5%; 95% CI: 0.06–13.1%) from the eastern area.

**Fig. 1:**
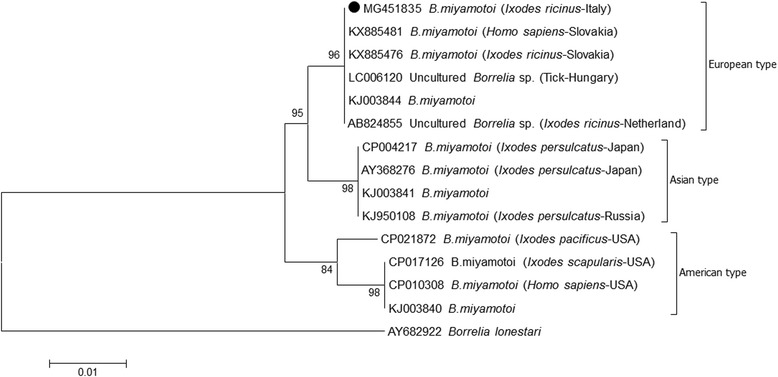
Phylogenetic tree of *glpQ* gene (625 bp) of *Borrelia miyamotoi*. Sequence dataset was analyzed using MEGA 6, the neighbour-joining (NJ) method, and bootstrap analysis (1000 replicates) based on the ClustalW algorithm. Significant bootstrapping values (> 70%) are shown on the nodes. *Borrelia lonestari* was used as an outgroup. The sequence generated in this study (MG451835) is indicated by a black circle

The two sequences obtained, one from the western area and one from the eastern area, on BLAST analysis both showed 100% identity with *B. miyamotoi* from the Netherlands (GenBank: AB824855) and 98% identity with *B. miyamotoi* from Japan (GenBank: CP004217). The longest sequence (888 bp) of this study was deposited in GenBank (MG451835) and used to perform the phylogenetic analysis (Fig. 1). The Italian sequence was identical (100% identity) to the sequences from the Netherlands, Slovakia and Hungary, and clustered within the European type.

## Discussion

Our findings are the first report of *B. miyamotoi* in *I. ricinus* ticks in Italy and indicate the presence of the pathogen across the Alpine arch. This is not surprising, since the pathogen has been reported to be widespread in questing *I. ricinus* in Europe and was also recently reported in southern European countries, i.e. Portugal [26], France [7] and Spain [27]. Our low prevalence is consistent with findings throughout Europe, where 1.8% of questing *Ixodes* ticks were found on average to be infected [8].

However, further study is needed to better define the distribution and prevalence of this pathogen in the vector and reservoir hosts in the Alps and other Italian regions where *I. ricinus* is present.

The eastern alpine area investigated in this study is a hot-spot of TBPs in Italy. Specifically, it accounts for the majority of human cases of Lyme borreliosis and tick-borne encephalitis [21, 22]. However, the risk of *B. burgdorferi* (*s.l.*) and other tick-borne infections is currently increasing in areas previously deemed unsuitable for *Ixodes* ticks, such as city parks [28], the Po River plain [29] and the western Alps [17].

Although no human cases of *B. miyamotoi* were unambiguously identified in Italy, misdiagnosis may have occurred at the time of other tick-borne infections causing fever. Indeed, consistent with the low prevalence in ticks, *B. miyamotoi* has been infrequently found in clinical human cases throughout Europe [5]. Relapsing fever, Lyme disease-like symptoms as skin rash, and human granulocytic anaplasmosis-like symptoms have been reported in human patients infected by *B. miyamotoi* [3, 30, 31].

## Conclusions

*Borrelia miyamotoi* has been shown to be a geographically widespread pathogen occurring at low prevalence in *I. ricinus* ticks in northern Italy. The focus on *Borrelia* genotypes causing Lyme disease has likely delayed this emerging pathogen being found in humans and vectors, and the use of specific tests or a metagenomic approach were pivotal to its discovery. Our results are intended to contribute to raising awarness of this pathogen amongst people in charge of TBP surveillance as well as in medical doctors, since unrecognized human *B. miyamotoi* infection may occur in areas endemic for *I. ricinus*.

## Abbreviations

TBP: Tick-borne pathogen
masl: Meters above sea level
PCR: Polymerase chain reaction
*glpQ*: Glycerophosphodiester phosphodiesterase gene
qPCR: Real time PCR
